# Accurate Identification and Mechanistic Evaluation of Pathogenic Missense Variants with *Rhapsody-2*

**DOI:** 10.1101/2025.02.17.638727

**Published:** 2025-02-22

**Authors:** Anupam Banerjee, Anthony Bogetti, Ivet Bahar

**Affiliations:** 1 Laufer Center for Physical and Quantitative Biology, Stony Brook University, New York 11794, USA; 2 Department of Biochemistry and Cell Biology, Renaissance School of Medicine, Stony Brook University, New York 11794, USA.

**Keywords:** Pathogenicity Prediction, Structural Dynamics, Missense Variants, Machine Learning

## Abstract

Understanding the effects of missense mutations or single amino acid variants (SAVs) on protein function is crucial for elucidating the molecular basis of diseases/disorders and designing rational therapies. We introduce here Rhapsody-2, a machine learning tool for discriminating pathogenic and neutral SAVs, significantly expanding on a precursor limited by the availability of structural data. With the advent of AlphaFold2 as a powerful tool for structure prediction, Rhapsody-2 is trained on a significantly expanded dataset of 117,525 SAVs corresponding to 12,094 human proteins reported in the ClinVar database. Adopting a broad set of descriptors, including evolutionary, structural, dynamic, and energetics features in the training algorithm, Rhapsody-2 achieved an AUROC of 0.94 in 10-fold cross-validation when variants of the same protein are not simultaneously included in the training and testing sets. Benchmarking against a variety of testing datasets demonstrated the high performance of Rhapsody-2. While evolutionary descriptors play a dominant role in pathogenicity prediction, structural dynamics features provide a mechanistic interpretation of the predicted effects, pathogenic or neutral, of SAVs. Notably, residues involved in allosteric communication, and those distinguished by pronounced fluctuations in the high frequency modes of motion or subject to spatial constraints in soft modes usually give rise to pathogenicity when mutated. Overall, Rhapsody-2 provides an efficient and transparent tool for accurately predicting the pathogenicity of SAVs and unraveling the mechanistic basis of the observed behavior, thus advancing our understanding of genotype-to-phenotype relations.

## Introduction

Missense mutations are genetic variations in which one or more nucleotide changes in the DNA lead to amino acid substitutions in the encoded protein. Unlike silent mutations, missense mutations can have a range of effects on the structure, function, and interactions of the encoded single amino acid variant (SAV). Pathogenic SAVs may exhibit severe disruptions in folding, enzymatic and other activities, and interactions with other proteins. These disruptions manifest as genetic diseases, cancer, and neurological disorders (1–4).

The human exome harbors a large number (76 million) of potential missense variants (5). Accurate prediction of the effects, pathogenic or benign, of these variants is a primary goal across clinical practice and public health. Such predictions may guide appropriate diagnosis and treatment strategies and facilitate disease prognostics and risk assessments, provided that they are made in the context of the evolution, structure, dynamics, and interactions of the mutants in the cell.

In recent years, many computational approaches powered by advances in machine or deep learning (ML or DL) and artificial intelligence (AI) techniques, as well as those employing robust traditional methods, have been developed for SAV pathogenicity predictions. These approaches include Missense3D (6), a homology modeling-aided structure-based predictor, SIFT (7), which uses sequence homology to compute the likelihood that an amino acid substitution will have an adverse effect on protein function; REVEL (8), an ensemble method that combines the results of multiple methods; EVE (9), a deep, generative model of evolutionary data; LYRUS (10), an ML predictor based on sequence, structure, and dynamics features; SPRI (11), a structure-based pathogenicity relationship identifier; PolyPhen-2 (12), an ML classifier enabled by high-quality multiple sequence alignments; and EVMutation (13), which leverages coevolution information to predict the fitness of mutants; WS-SNPs&GO (14), a server that uses sequence, structure, and GO annotations to predict diseases associated with SNPs; and MutPred (15), an early predictor of pathogenicity, incorporated both functional and structural properties, such as catalytic activity and post-translational modifications, to estimate disease mechanisms. The updated MutPred2 (16) includes a wide range of properties, including secondary structure, signal peptides, and transmembrane topology. MVP and ESM1b are state-of-the-art tools for predicting missense variant pathogenicity. MVP uses a supervised deep residual network trained on labeled pathogenic and benign variants, integrating evolutionary, structural, and gene-specific features (17). In contrast, ESM1b, an unsupervised protein language model, predicts effects across ~450 million missense variants using sequence-based representations, excelling in isoform-specific and complex variant analyses like in-frame indels (18).

A recent addition to this arsenal is AlphaMissense (19), an *ab initio* deep-learning method, relying on evolution information, which fine-tunes AlphaFold (20) predictions of human and primate variant population frequency databases. Of more than 71 million missense variants in the human proteome, 32% are classified by AlphaMissense as pathogenic, 57% as benign, and 11% as variants of uncertain significance (19). AlphaMissense integrates sequence and structural context by combining co-evolutionary insights from multiple sequence alignments with AlphaFold-derived structural embeddings. It models sequence context using unsupervised protein language modeling and masked residue prediction to capture amino acid distributions and evolutionary constraints. Structural context is encoded through AlphaFold’s pair representations and positional embeddings, processed via Evoformer (20) layers to align sequence and structure features. Fine-tuning on population frequency data enables robust pathogenicity predictions across the proteome, including rare or novel variants, while maintaining high precision in both structured and disordered regions.

These tools have driven remarkable advances in the field. Yet, accurate prediction of pathogenicity by itself is not sufficient for understanding the mechanistic basis of observed behavior, nor does it provide insights into the design of rational intervention strategies. As recently pointed out (21), the merger of ML/AI methods with those based on physical sciences holds great promise for gaining a deeper understanding of target proteins’ structural dynamics at multiple scales; and structural dynamics, in turn, defines the mechanisms of motions accessible to achieve biological function and interactions, whose disruption (due to point mutations at critical sites) may cause dysfunction. The need for considering dynamic effects to understand pathogenicity may extend to tissue-specific non-Mendelian mutations, as suggested by the examination of EGFR ectodomain mutations on kinase domain activation (22). A combined approach that mutually benefits from advances in ML/AI methodology and fundamental theory and concepts of structural biophysics can help provide accurate estimates of pathogenicity *and* shed new insights into the molecular basis of ML/AI predictions, thus potentially accelerating the discovery of intervention methods. The current study aims at achieving those two goals.

The integrated approach between the two disciplines (ML/AI and biophysics) needs a biophysical model and method that lends itself to high throughput generation of data (at the proteome scale), which may then be used for training ML models. Elastic network models (ENMs) meet this requirement. The simplicity of ENMs allows generating mathematically exact analytical solutions, uniquely defined by the protein architecture, for the collective modes of motion intrinsically accessible to the structure, which often underlie biological function.

In 2018, we developed a dynamics-dependent ML-based predictor of SAV pathogenicity (23) and implemented it in the interface *Rhapsody* (24). *Rhapsody* used as features not only sequence and structure data, but also ENM-based descriptors of protein dynamics. Strikingly, despite the simplicity of the approach (a random forest algorithm with only ten features, including three sequence-dependent, one structure-dependent and four dynamics-dependent), the method, benchmarked against a dataset of about 20,000 annotated variants, outperformed the (then) state-of-the-art methods for classifying SAVs as deleterious/pathogenic or neutral/benign. Given that the major difference with respect to existing tools was the incorporation of protein dynamics, we emphasized that the latter was an important determinant of the impact that missense mutations have on protein function. Since then, *Rhapsody* has been widely used as a sequence-, structure- and dynamics-dependent predictor of pathogenicity, with sequence-specific properties being mainly provided by PolyPhen-2 (12).

Yet, the application of *Rhapsody* depends on the availability of a known structure for the protein of interest. This requirement limited the size of the training set as well as the application to structurally unresolved proteins. Precisely, despite identifying an integrated dataset of 87,726 SAVs by combining five publicly available datasets (25–29), only 27,655 SAVs could be matched with PDB structures (23), and *Rhapsody* was trained on an even smaller dataset of 20,361 SAVs corresponding to 2,423 unique human proteins (24) after selecting the SAVs with sufficiently high-confidence labels in ClinVar (2) .

With the availability of structural data for most human proteins made possible by AlphaFold2 (20), we are in a position to address the above limitation. By leveraging the available wealth of structural data accessible in the AlphaFold database, as well as recent advances in ENM methodology and ML algorithms, we present here *Rhapsody-2,* a predictor with significantly higher accuracy and coverage than its predecessor. Importantly, *Rhapsody-2* also provides insights into the mechanistic basis of the predictions. The new training database (*Rhapsody-2* DB) comprises 117,525 SAVs spanning 12,094 human proteins; and the new algorithm considers 100 descriptors, composed of 22 evolutionary, 17 structural, 21 dynamics-based, 33 energetics-based, and 6 physicochemical (residue-specific) features, and one on intermolecular interactions (Supplemental Information (SI) Table S1).

Accurate evaluation of pathogenicity prediction tools requires addressing biases that occur when variants from the same protein are included in both training and testing datasets, leading to inflated performance metrics (30). To avoid this, *Rhapsody-2* was evaluated using protein-stratified cross-validations, ensuring that variants from the same protein were confined to either the training or testing sets. Under these conditions, *Rhapsody-2* achieved an AUROC of 0.94, reflecting strong predictive performance. In addition, when tested on independent benchmark datasets, all proteins present in the test sets were removed from the training data to prevent overestimation of accuracy. Even with this careful separation, *Rhapsody-2* performed comparably to state-of-the-art tools that do not implement such exclusions, underscoring its robustness and generalizability. These results address our first objective of delivering a reliable pathogenicity predictor, while our second objective—understanding the biophysical basis of pathogenicity—is met by analyzing feature distributions associated with deleterious and neutral mutations. Overall, *Rhapsody-2* provides both accurate predictions of single amino acid variant (SAV) pathogenicity and insights into their molecular mechanisms, advancing our understanding of genotype-phenotype relationships.

## Results

### *Rhapsody-2* database, features, and methodology.

**Figure 1.**
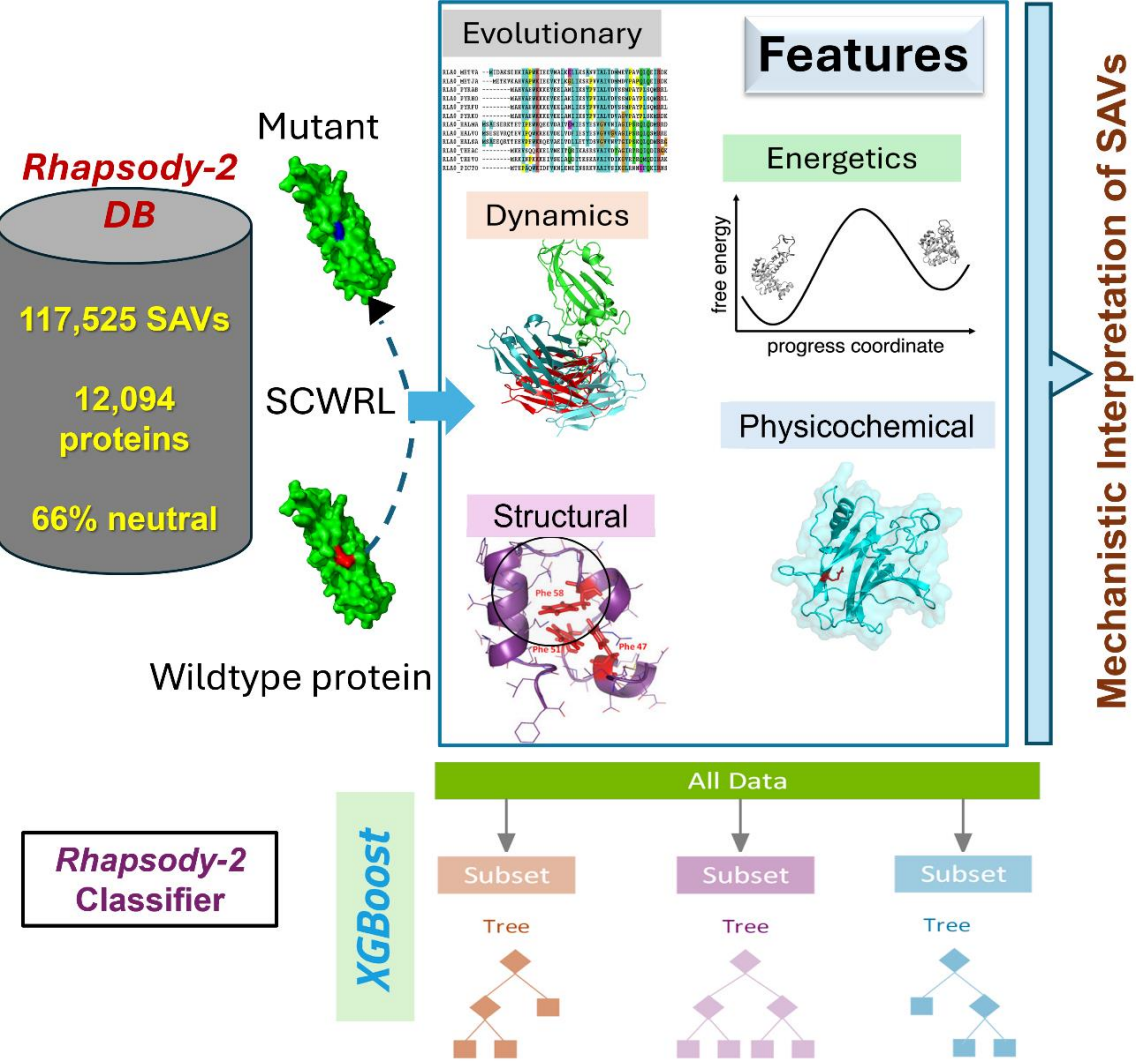
Schematic description of *Rhapsody-2* inputs, method, and outputs. For each SAV in *Rhapsody-2* DB, 100 descriptors composed of 6 residue physicochemical properties, 17 accounting for structural properties, 22 based on sequence evolution, 33 based on energetics, 21 based on intrinsic dynamics, and 1 based on intermolecular interactions were computed. Complete or partial sets of descriptors were used in training *Rhapsody-2* classifier or its variants, respectively, with XGBoost algorithm. Each point mutation was modeled using SCWRL. The distributions of descriptor values in pathogenic and neutral SAVs were used to make inferences on the mechanistic basis of predictions.

Table 1 provides a summary of the advancements in *Rhapsody-2*, compared to the original *Rhapsody*. Figure 1 provides a schematic description of the methodology. Among the five categories of features used in *Rhapsody-2*, structural features measure how the mutation is accommodated in the folded structure, including the overall packing and interactions with spatial neighbors. Physicochemical features refer to the specific properties of amino acids in the wild-type (WT) protein and its SAV. Dynamics features refer to the spectrum of equilibrium (collective) motions and allosteric communication properties shortly called intrinsic dynamics, evaluated using two elastic network models (ENMs) implemented in the *ProDy* interface (31, 32): the Gaussian network model (GNM) (33) and the anisotropic network model (ANM) (34, 35). Evolutionary features include conservation and the potential tolerance to single or compensating amino acid variations across sequential homologs using the DIAMOND package (36). Additionally, we assessed the energetic properties of the folded state for both the WT and mutant protein. Detailed descriptions of these attributes are provided in the SI Methods.

Gradient boosting is a machine learning technique that builds models iteratively by adding decision trees, with each tree correcting the errors of the previous ones. XGBoost (eXtreme Gradient Boosting) enhances this method with second-order gradient optimization for precise loss minimization, L1/L2 regularization to prevent overfitting, sparsity-aware algorithms for missing data, and efficient tree pruning for better generalization (37). Its scalability and parallel execution make it highly effective for high-dimensional, structured datasets. We used the XGBoost framework (37) to construct different variants of the *Rhapsody-2* classifier trained on all or a subset of features, including one model exclusively based on intrinsic dynamics.

### Evaluation of the performance of *Rhapsody-2*.

**Figure 2.**
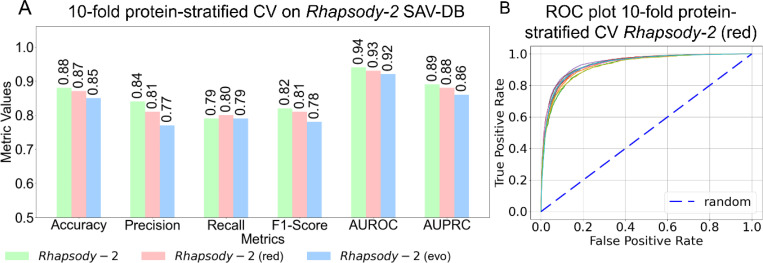
Performance of Rhapsody-2 and its variants under protein-stratified 10-fold cross-validation. **(A)** Performance of *Rhapsody-2* and its variants evaluated using 10-fold protein-stratified cross-validation. The full model and its variants are indicated by different colors (see color code at the bottom). All threshold-dependent metrics are based on a classification threshold of 0.5. **(B)** ROC curves for the reduced model *Rhapsody-2* (red) evaluated under 10-fold protein-stratified cross-validation. The dashed line indicates random behavior.

Recognizing the potential bias toward accurate prediction when different variants of the same protein appear in both training and testing datasets, as highlighted by Grimm et al. (30), we evaluated *Rhapsody-2* performance using, as metrics, accuracy, precision, recall, F1-score, AUROC and AUPRC (see definitions in SI Methods), averaged over 10-fold protein stratified cross-validations on *Rhapsody-2* DB. In these tests, we removed from the training set all variants of a given protein if a SAV (of the same protein) is in the test set. As shown in Figure 2A, Rhapsody-2 achieved an AUROC of 0.94 and an AUPRC of 0.89 using 10-fold protein-stratified cross-validation. In contrast, the results without protein stratification (Figure S1) show higher AUROC and AUPRC values of 0.97 and 0.94, respectively, suggesting inflated performance due to the overlap of proteins between the training and testing folds.

### Performance of *Rhapsody-2* variants trained on subsets of descriptors.

We further examined the performance of *Rhapsody-2* variants, trained on selected subsets of descriptors to assess the contribution of different categories of descriptors to pathogenicity prediction. The *Rhapsody-2(struct)* variant trained exclusively on structural descriptors (listed in rows 77–93 of SI Table S1) exhibited a mean AUROC of 0.87 under *protein-*stratified 10-fold cross-validation. *Rhapsody-2* (dyn), a model trained solely on dynamics descriptors (rows 23–43 of Table S1), achieved an AUROC of 0.79. Note that the dynamics descriptors are based on ENM analyses which exclusively refer to the structure-encoded intrinsic dynamics of the WT protein. These descriptors are agnostic to amino acid identity and are fully defined by the WT inter-residue contact topology. Finally, we tested the performance of *Rhapsody-2(evo)*, a model trained using evolutionary descriptors only (rows 1–22 in Table S1). *Rhapsody-2(evo)* achieved an AUROC of 0.92 and AUPRC of 0.86 under 10-fold protein stratified CV, confirming that sequence evolutionary information is highly predictive, as also shown by other methods such as *EVE*.

These experiments show that alternative models with comparable performances can be built with different categories of descriptors (each containing around 20 features). They further indicate that the different categories of descriptors carry interdependent or partially redundant information. This is consistent with the correlation between sequence evolution and structural dynamics pointed out earlier (38). However, the performance of these variants remains below that of *Rhapsody-2*. Next we explored whether a reduced model could be constructed with a limited set of descriptors without significantly compromising on the classification ability of the model.

### Construction of *Rhapsody-2(red)*, a reduced model trained on a small set of descriptors.

Our *in silico* experiments in search of a reduced model led to *Rhapsody-2(red)*, trained on two categories of descriptors, evolutionary and dynamics, plus one structural feature, the relative solvent accessibility (RSA). Despite the use of a reduced set of 44 descriptors, this model exhibited a performance almost as strong as the full *Rhapsody-2* (see the *blue* and *pink bars* in Figure 2A), and even surpassed *Rhapsody-2* in terms of its recall obtained by protein-stratified CV. Figure 2B present the ROC plots for *Rhapsody-2(red)* under 10-fold protein stratified CV. *Rhapsody-2(red)* is therefore proposed as a simple and computationally efficient tool: with precomputed data on ENM-based dynamics and evolutionary descriptors, and RSA available for almost all human proteins, it generates *in silico* saturation mutagenesis maps within seconds.

### Benchmarking the performance of *Rhapsody-2* against state-of-the-art predictors of pathogenicity.

**Figure 3.**
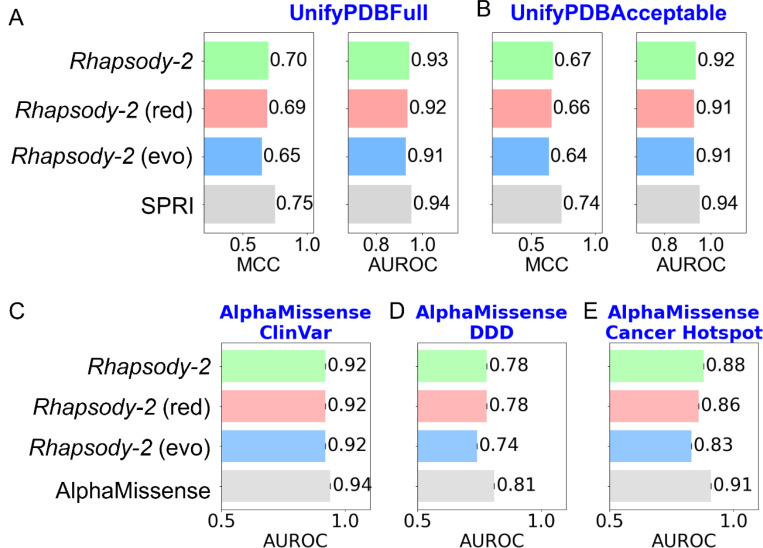
Rhapsody-2 demonstrates performance comparable to benchmark classifiers while ensuring no redundant proteins between training and test sets. **(A)** and **(B)** show results for UnifyPDBFull and UnifyPDBAcceptable datasets, evaluated using protein-stratified 5-fold cross-validation. **(C)**, **(D)**, and **(E)** present AUROC metrics for AlphaMissense datasets, specifically ClinVar, DDD, and Cancer Hotspot, respectively. Classifiers were trained on the *Rhapsody-2* SAV database, rigorously curated to exclude redundant proteins across training and evaluation datasets.

First, we considered the two datasets UnifyPDBFull and UnifyPDBAcceptable, both introduced alongside the SPRI method. Note that neither SPRI nor the other methods used for comparison were benchmarked using protein-stratified cross-validations against these datasets. To enable a direct comparison between our method, SPRI, and the other methods, we trained *Rhapsody-2* and its variants on UnifyPDBFull and UnifyPDBAcceptable datasets (using the XGBoost algorithm) and adopted the same 5-fold cross-validation protocol introduced alongside the SPRI method (11). The results are presented in Table S2. *Rhapsody-2* achieved an average AUROC of 0.96 and an average Matthews Correlation Coefficient (MCC) of 0.78 on the UnifyPDBFull benchmark, and respective values of 0.95 and 0.76 on UnifyPDBAcceptable. The next best method, SPRI (11) , had an AUROC of 0.94 and an MCC of 0.75 on UnifyPDBFull, and 0.94 and 0.74 on UnifyPDBAcceptable. SPRI was shown to outperform PROVEAN (39), PolyPhen-2 (12), PMUT (40), LIST (41), FATHMM (42), EVmutation (13), and the original *Rhapsody* against both datasets (11). However, as previously observed, the inclusion of SAVs from the same proteins in both training and testing folds leads to inflated performance metrics. To address this and ensure more stringent and rigorous benchmarking, we present the results of 5-fold protein-stratified cross-validation for Rhapsody-2 and its variants in Figures 3A and 3B, using the UnifyPDBFull and UnifyPDBAcceptable datasets, respectively. For comparison, results for SPRI—without protein stratification—are also included.

*Rhapsody-2* achieved an average AUROC of 0.93 and an MCC of 0.70 on the UnifyPDBFull dataset, while *Rhapsody-2* (red) recorded an AUROC of 0.92 and an MCC of 0.69. Similarly, on the UnifyPDBAcceptable dataset, *Rhapsody-2* achieved an AUROC of 0.92 and an MCC of 0.67, with *Rhapsody-2* (red) slightly lower at 0.91 AUROC and 0.66 MCC. These results highlight the robust classification performance of *Rhapsody-2* and its variants under protein-stratified conditions.

Next, we benchmarked *Rhapsody-2* models using three independent test sets: AlphaMissense ClinVar, Deciphering Developmental Disorders (DDD) and Cancer Hotspot (19). As AlphaMissense (and other methods compared with it) did not exclude proteins present in the test sets from the training data, this introduces the possibility of data leakage. However, to enable a fair comparison between Rhapsody-2, its variants, and AlphaMissense, we adhered to two testing conditions. First, (a) we trained Rhapsody-2 and its variants on our Rhapsody-2 DB, ensuring that the SAVs from the AlphaMissense test sets were not included in the training sets; and (b) we trained Rhapsody-2 and its variants on subsets of Rhapsody-2 DB that excluded all proteins whose SAVs were present in the three corresponding AlphaMissense test sets. This resulted in reduced training datasets of 65,382, 107,955, and 110,085 SAVs for benchmarking the corresponding Rhapsody-2 models and their variants against the AlphaMissense ClinVar, DDD, and Cancer Hotspot datasets, respectively. The results from the tests *(a)* and *(b) are* presented in Table S3. As described in more detail below, the two sets of tests led to AUROC values that strongly support the high performance and robustness of Rhapsody and its variants *Rhapsody-2(red)* and *Rhapsody-2*(*evo*).

Figure 3 (C–E) and Table S3 provide detailed AUROC values from test (*b*), demonstrating the robustness and consistency of *Rhapsody-2* and its variants across multiple datasets. For the ClinVar dataset, *Rhapsody-2* achieved an AUROC of 0.916, while *Rhapsody-2* (red) and *Rhapsody-2* (evo) recorded AUROCs of 0.920 and 0.917, respectively. In the DDD dataset, Rhapsody-2 achieved an AUROC of 0.781, with *Rhapsody-2* (red) at 0.779 and Rhapsody-2(evo) at 0.741. For the Cancer Hotspot DB, *Rhapsody-2* attained an AUROC of 0.878, with *Rhapsody-2(red)* at 0.861 and *Rhapsody-2* (evo) at 0.833. The strong performance of *Rhapsody-2* and its variants in test (*b*), closely matching that of AlphaMissense despite the exclusion of same-protein data, underscores its robustness, generalizability, and practical applicability across diverse datasets.

To ensure a fair comparison with AlphaMissense and other related methods, Panels A-C in Figure S2 present the results from the respective datasets—ClinVar, DDD, and Cancer Hotspot DBs—for test (a). The mean AUROC reported for *Rhapsody-2* in panel **A** is 0.917 (from 1,000 bootstrap resamples); and those of *Rhapsody-2(evo)* and *Rhapsody-2(red)* are both 0.926. These values are lower than that of AlphaMissense (0.940) but surpass that (0.911) of EVE (9). EVE was the second-best performing method in previous benchmark against Missense ClinVar DB, with AUROC higher than those of Eigen (43), CADD (44), PolyPhen-2_HVAR (12), ESM1b (45), SIFT (46), PolyPhen-2_HDIV (12), ESM1v (47), and PrimateAI (48), in that order. Figure S2B shows that *Rhapsody-2’s* performance (0.800) is comparable to AlphaMissense (0.809) and the next best performer PrimateAI (0.797) when benchmarked against DDD. In this case, PrimateAI was found to outperform PolyPhen-2_HVAR, ESM1b, PolyPhen-2_HDIV, VARITY_R_LOO (49), gMVP (50), REVEL (8), Eigen, CADD, ESM1v, and SIFT. Finally, Figure S2C shows that the performance of *Rhapsody-2* (0.896) is close to that AlphaMissense (0.907), and higher than the next best method, VARITY (49), when benchmarked against the Cancer Hotspot DB. VARITY outperformed gMVP, REVEL, ESM1b, EVE, ESM1v, CADD, Eigen, PolyPhen2_HVAR, PrimateAI, PolyPhen2_HDIV and SIFT in that order.

We note that all these methods, except AlphaMissense, have been trained on limited data available at the time they were developed. Therefore, their relatively lower performance may be due to their training with more limited data.

### Features contributing maximally to classification provide insights into the rationale for SAV predictions by *Rhapsody-2*.

**Figure 4.**
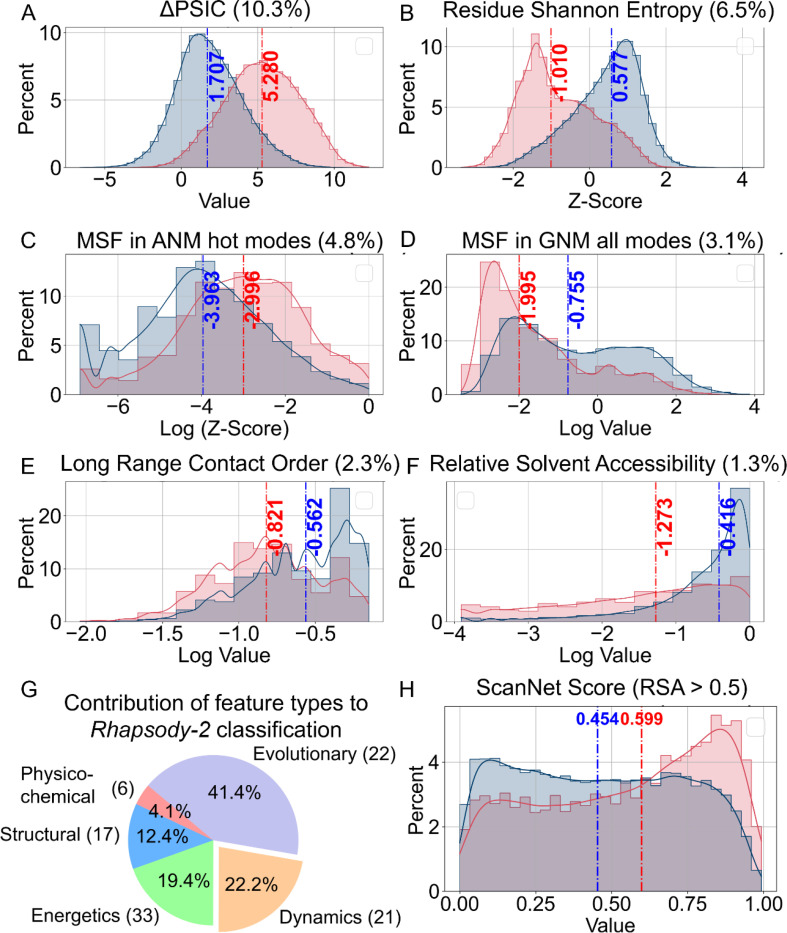
Distribution of selected features distinguished by their high discriminatory power between neutral and pathogenic SAVs, and contributions of five groups of features to Rhapsody-2 predictions. **(A-F)** Histograms of top contributing features for neutral (*blue*) and deleterious (*red*) mutations, shown for **(A-B)** evolutionary, **(C-D)** intrinsic dynamics, **(E-F)** local interactions features. Median values are indicated by dotted lines. The abscissa in **C-F** are in log scale for clearer visualization. **(G)** Percent contributions of different groups of features to *Rhapsody-2* protein-stratified 10-fold classification (see Table S1). **(H)** Distributions of ScanNet values (predicted probabilities of lying at intermolecular interfaces) for *Rhapsody-2* DB subset of SAVs (48,909 of them) that are solvent exposed (RSA > 0.50).

The relative contributions of individual features to the *Rhapsody-2 model (*SI Table S1) shed light on the biophysical origins of the predictions. A closer look reveals the features that play a dominant role in determining the effect of the mutation. Notably, among the 100 features, the difference in Position-Specific Independent Counts score (ΔPSIC) (51) between the WT and mutant residue makes the largest contribution (10.30%) to classification, the values corresponding to neutral and pathogenic mutations showing distinctive distributions (respective *blue* and *red* histograms with medians of 1.71 and 5.28 in Figure 4A). PSIC reflects the evolutionary conservation at a given position. Its large contribution to *Rhapsody-2* predictions is consistent with the significant effect of replacing a conserved residue. The second most important feature among evolution-related features is the z-score of the Shannon Entropy of the WT residue at the substitution site. This feature contributes 6.54% to classification (Figure 4B). It measures the similarity between the WT residue at the substitution site and those of its sequential homologs. A median of −1.01 (highly dissimilar) versus 0.58 (similar) distinguishes the pathogenic and benign variants. Sequence conservation is closely followed by size-based conservation with 5.3% contribution (see SI Table S1).

Among the features describing the protein dynamics, two stand out: participation in high frequency modes of motions, as predicted by the anisotropic network model (ANM) (34), and mean-square-fluctuations (MSFs) under equilibrium conditions, predicted by the Gaussian Network Model (GNM) (33) (Figure 4C–D). The z-score associated with the former contributes 4.84% to classification. High-frequency modes are manifested by localized fluctuations at the most tightly packed (core) regions, known as kinetically hot sites; these often act as folding nuclei and tend to underlie stability (52–54), hence their strong resistance to substitutions (55). The GNM MSF at the mutation site, on the other hand, contributes 3.13% to classification, ranking second among dynamics features. Its higher median for benign variants, compared to that for pathogenic, shows that mutations at sites that are highly mobile (large MSFs) are more likely to accommodate substitutions, and *vice versa*.

We further show in Figure S3 that residues that tend to act as effectors of allosteric signals are more likely to give rise to pathogenicity if mutated. Both GNM- and ANM-based predictions corroborate this behavior. This effect is particularly strong when focusing on GNM soft modes (*upper left panel*). Note that soft (lowest frequency) modes of motion usually entail highly cooperative rearrangements embodying most, if not all, of the structure. It is conceivable that mutations at residues acting as effectors of allosteric signals in the most cooperative modes are prone to cause pathogenicity. Likewise, the residues distinguished by high spatial cross-correlations (positive or negative) with other residues (*bottom right*) show high tendency to give rise to pathogenicity, if mutated.

As to the 17 features associated with local interactions, the most influential is the long-range contact order (LRCO) and relative solvent accessibility (RSA) of the WT residue (Figure 4E–F). LRCO quantifies the extent of long-range inter-residue contacts. Contacts are defined as those between residues separated by at least three intervening amino acids along the sequence with farthest atom-atom distance of 8 Å. The LRCO values are normalized with respect to the total number of possible contacts within this distance range. Pathogenic variants exhibit a median LRCO of 0.40, indicating a higher propensity of long-range interactions compared to benign variants (whose median LRCO is 0.18), i.e., amino acids engaged in a higher number of contacts are more susceptible to pathogenicity due the disruption of their extensive interactions. Finally, mutations at buried sites, characterized by low RSA, often disrupt core interactions and structural stability, making them less tolerant to mutations (Figure 4F). Table S4 lists the median values of each feature for neutral and pathogenic SAVs, along with their variance and statistical significance.

The pie chart in Figure 4G highlights the relative contributions of different categories of descriptors to *Rhapsody-2* XGBoost classifier, under protein-stratified cross-validation. The evolutionary features make a dominant contribution of 41.4%, followed by dynamics (22.2%) and energetics (19.4%). Note that the model *Rhapsody-2(red)* excluded the energetics, physicochemical and structural descriptors (except RSA) with minimal loss of predictive power. Therefore, the contribution of different descriptors is model-specific.

Finally, ScanNet (56) (the sole feature representing intermolecular interactions and contributing ~0.5% to classification) was used to evaluate the probability of WT residues lying at an interface for the examined sites of mutations. Figure 4H displays the histograms of ScanNet scores corresponding to pathogenic (*blue*) and benign (*red*) subsets of SAVs, using as reference sufficiently exposed (RSA > 0.5) mutation sites. Pathogenic variants have a median score of 0.60, while benign variants score 0.45 (Figure 4H), indicating that solvent-exposed residues with higher propensity to make intermolecular contacts tend to cause pathogenicity when mutated.

### Impact of pLDDT Thresholding on Variant Classification and Feature Contributions.

**Figure 5:**
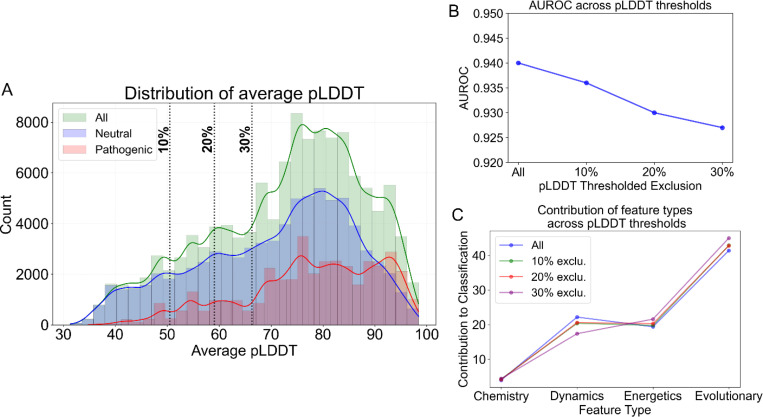
The impact of pLDDT thresholding on classification performance and feature contributions. **(A)** Distribution of average pLDDT scores across all variants (green), neutral variants (blue), and pathogenic variants (red). Kernel density estimates are overlaid on histograms. Vertical dashed lines indicate pLDDT exclusion thresholds at 10%, 20%, and 30%. **(B)** AUROC across pLDDT threshold exclusions, showing a decline in classification performance as lower-confidence residues (low pLDDT) are progressively excluded. **(C)** Contribution of different feature types (Chemistry, Dynamics, Energetics, Evolutionary) to classification performance at different pLDDT threshold levels. While contributions remain relatively stable, there is a slight decrease in the importance of Dynamics features and a corresponding increase in the contribution of Evolutionary features with more stringent pLDDT thresholds.

The distribution of average pLDDT scores (Figure 5A) suggests that neutral single amino acid variants (SAVs) are more common in proteins with lower predicted structural confidence compared to pathogenic SAVs. Lower pLDDT values (pLDDT < 50) can indicate either intrinsically disordered or flexible proteins or structurally predictable proteins with lower model confidence. In Figure 5A, we show that neutral variants tend to occur in proteins with lower average pLDDT. Similarly, Figure S4 (bottom panel) examines pLDDT at the mutated residue level, showing that neutral variants have a lower median residue pLDDT (69.04) than pathogenic ones (92.21). This suggests that mutations in flexible or structurally uncertain residues are more likely to be tolerated, whereas those in higher-confidence regions may have stronger structural or functional impacts.

As we progressively increased the average pLDDT threshold of the protein by excluding the lowest-confidence proteins—removing the last 10% (Average pLDDT 50.52), 20% (59.09), and 30% (66.31)—we observed a slight decrease in AUROC (Figure 5B). This decline is likely due to a reduction in the number of training instances, as proteins with lower pLDDT were excluded.

On observing the feature contributions in Figure 5C, we note that the overall ranking of feature importance remains largely consistent across thresholds. Evolutionary features, which are entirely sequence-based, continue to contribute maximally to pathogenicity prediction, followed by dynamics-based features that depend on the protein’s structural topology. There is a slight decrease in the contribution of dynamics-based features as the pLDDT threshold increases, accompanied by a proportional increase in the importance of evolutionary and energetics-based features. This trend could be attributed to the exclusion of more flexible proteins at higher thresholds, reducing the impact of dynamics-driven attributes, while the improved structural reliability enhances the contribution of energetics-based factors.

Given that pathogenicity prediction relies on multiple feature types, with the highest contributor being sequence-based evolutionary features, we prefer to consider the full dataset without filtering based on pLDDT. This ensures a broader representation of SAVs across diverse human proteins while maintaining a larger dataset, which is advantageous for model robustness and generalizability.

### A case study illustrating the interpretability of Rhapsody-2 predictions.

**Figure 6.**
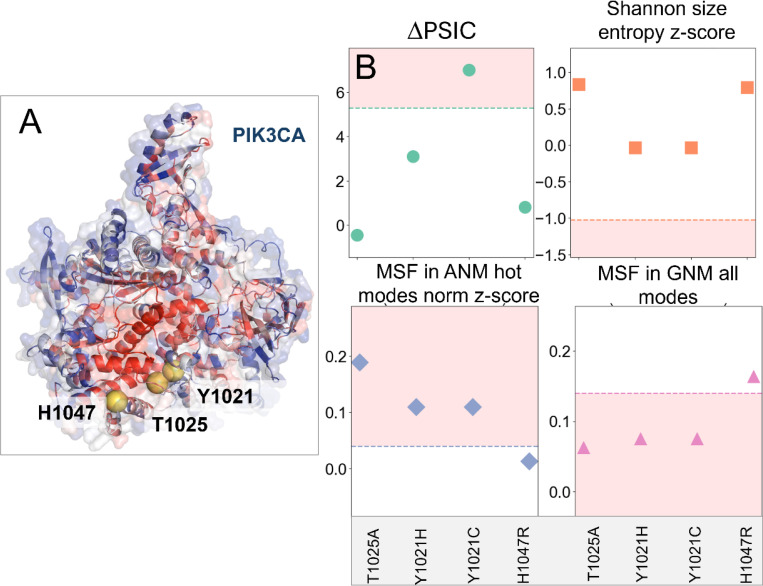
Evaluation of the origin of pathogenicity based on the SAV features examined with respect to median values for pathogenic behavior, illustrated for PIK3CA four mutations. **(A)** Ribbon diagram of PIK3CA color-coded by its residue-averaged pathogenicity profile as predicted by *in silico* saturation mutagenesis heatmap (Figure S5) generated by *Rhapsody-2* (*red*). The (average) pathogenicity values range from 0 (neutral in *blue*) to 1(pathogenic in *red*). The mutated residues are shown in *yellow spheres*. **(B)** Key evolutionary and dynamics features (ordinate) evaluated for the four mutations (abscissa) reported to be pathogenic in ClinVar. Median values of these features (for all DB SAVs) delimit the regions shaded in *pink*, that are strongly pathogenic. Intrinsic dynamics (*bottom two plots*) play a significant role in explaining the pathogenicity of these mutations.

To illustrate how the outputs *from Rhapsody-2* can help make inferences on the origin of pathogenicity, we present a case study, mainly four pathogenic variants reported for phosphatidylinositol 4,5-bisphosphate 3-kinase catalytic subunit a (PIK3CA) (Figure 6A) in the AlphaMissense ClinVar dataset. The α-subunit of PIK3s assumes different conformations in inactive and active forms, which are essential to its catalytic activity (56). The C-terminal end of the catalytic domain of PIK3s “shields” the ATP binding site and is thought to play a role in regulating catalysis, by undergoing a significant conformational change (57).

*Rhapsody-2*(*red*) accurately predicts all four mutations T1025A, Y1021H, Y1021C, and H1047R to be pathogenic. Figure 6B displays the values of four dominant features (ΔPSIC, size entropy z-score, MSF in ANM high frequency modes and MSF in all GNM modes) for these mutations, compared to the median values for all pathogenic SAVs (Table S4). The regions shaded in *pink* indicate the pathogenic regions, based on the medians used as threshold. Three out of four mutants have ΔPSIC values below the median of 5.28 (see Figure 6A), indicating that ΔPSIC was influential only for one of the mutations (Y1021C) and not for the other three. Likewise, the size-based Shannon entropies of the four mutants do not lie within the pathogenic region, indicating that the size change was *not* a factor underlying the observed (and predicted) pathogenicity. Notably, the MSFs in both the ANM hot modes and the GNM all modes show that T1025A, Y1021H, and Y1021C lie in the pathogenic regime, pointing to the role on intrinsic dynamics in pathogenicity prediction. As to the fourth mutation, H1047R, other features appear to have determined the fate of this mutation, while proximity to the pathogenicity median suggests that these properties may also have contributed to the classification.

## Discussion and Conclusion

The goal of the present study was 2-fold: to deliver a comprehensible and transparent tool for accurately predicting the pathogenicity of SAVs using not only ‘statistical’ data on sequence patterns but also biophysical data on structure, dynamics and interactions; and to provide a platform for mechanistic interpretation of the predictions based on these biophysical features. *Rhapsody-2* meets both goals. It is a comprehensible tool that provides insights into the biophysical basis of disease-causing mutations. Its ability to integrate and analyze diverse data helps gain a deeper understanding of the functional effects of mutations and develop more effective therapies. A few noteworthy observations are in order.

### High performance of predictors based exclusively on dynamics- or structure-descriptors.

A model trained on 21 dynamics-based descriptors only, *Rhapsody-2(dyn)*, achieved a decent performance (AUROC: 0.79) in distinguishing between pathogenic and neutral variants. *Rhapsody-2(struct)* variant trained exclusively on 17 structural descriptors also exhibited a mean AUROC of 0.87 on 10-fold protein-stratified cross validation. We found that dynamics-based descriptors play a significant role in pathogenicity prediction, as the second highest contributor (22.2%) to *Rhapsody-2*, after sequence evolutionary descriptors (41.4%); whereas structural descriptors contribute by 12.4%.

### Dominant role of evolutionary features.

The *Rhapsody-2(evo)* variant exclusively based on sequence-evolutionary descriptors performed better (AUROC of 0.93, which decreased to 0.92 only upon protein-stratification) than structure- and dynamics-based variants, reminiscent of the success of other sequence-based models such as EVE (9) and EVmutation (13). This observation further points to the interdependence of the descriptors grouped in different categories originating from the fundamental tenets: sequence determines structure, which in turn defines the equilibrium dynamics or the collective motions predicted by ENMs. Our earlier large-scale analysis (38) also showed how sequence evolution goes hand in hand with structural dynamics: e.g., evolutionarily conserved regions exhibit minimal displacements in the global modes and maximum fluctuations in local modes (52); whereas sequentially variable regions are structurally variable too. Not surprisingly, the fluctuations at the high frequency end of the ANM spectrum turn out to be the most influential feature among dynamics-based descriptors (Table S1); residue undergoing such motions are intolerant to mutations and evolutionarily conserved.

### Utility of training on diverse groups of descriptors.

A reduced model, *Rhapsody-2(red),* based on evolutionary and dynamics features outperformed both *Rhapsody-2(evo)* and *Rhapsody-2(dyn).* This indicates that these descriptors, despite notable overlaps, do contain complementary information and there is a merit to consider both sets of predictors as illustrated in Figure 6. Besides, statistically observed evolutionary patterns can be explained by structure or dynamics features, which may help develop rational design or intervention strategies. *Rhapsody-2* permits us to assess the origin of the predictions. To this aim, one may refer to the histograms of dominant features generated for neutral and pathogenic SAVs, or the tabulated median values of all features for neutral and pathogenic mutations and examine how the individual SAV’s features compare to those data.

### Availability of AlphaFold DB.

The superior performance of *Rhapsody-2* (and AlphaMissense) compared to other pathogenicity predictors can be largely attributed to the vast amount of structural data made available through AlphaFold DB. AlphaMissense and ESM1b indeed represent a paradigm shift by leveraging advanced sequence and structural data, which earlier methods did not benefit from. The other methods could have shown comparable performance if they had access to the same data.

### Pushing the limits of current technology to address specialized and/or context dependent behavior.

The current results (AUROC of 0.94 in *protein-stratified* cross-validations) suggest that we are approaching the limits of what can be achieved with existing data, given possible errors or uncertainties in the data themselves. Yet, there are still areas that demand further work, especially in the case of specialized datasets. For example, *AlphaMissense* surpassed *Rhapsody-2* by less than 2% when tested against DDD but their predictive performance, as measured by AUROC, remained below 0.81. It may be necessary to develop more system-specific classifiers, such as those customized to membrane proteins, or multimeric proteins, or manifested in different cell/tissue contexts. *Rhapsody-2* and most pathogenicity predictors are based on the properties of the SAVs themselves without explicit consideration of systems/context-dependent effects. Yet, the interactions in the cellular environment and how the mutations impact those interactions may be a serious consideration, and there are recent studies that focus on the impact of mutations on protein-protein interaction interfaces (57, 58).

### Structural confidence influences variant classification, but full data inclusion ensures robustness.

Our findings show that neutral variants are more prevalent in proteins with lower pLDDT scores, indicating greater tolerance in flexible or structurally uncertain regions. While increasing pLDDT thresholds slightly reduced AUROC, feature contributions remained consistent, with evolutionary features dominating. To maximize model robustness and generalizability, we opted to include the full dataset without pLDDT-based filtering.

### Other challenges.

Characterization of the effects of multiple mutations, insertion/deletions of loops, segments, association/dissociation of entire domains/subunits, and intrinsically disordered protein (IDP) segments remain as challenging tasks that await further work. In addition, post-translational modifications, transmembrane topology and small-molecule, metal or ion binding, are crucial to function (15, 16, 59), which may need attention in future pathogenicity predictors. We have not explicitly analyzed intrinsically disordered protein (IDP) regions during the training or evaluation of *Rhapsody-2*. Yet, the pLDDT score distributions in Figure S4 show that SAVs from IDP regions are included in the *Rhapsody-2* database. Given the functional significance of IDPs in cellular context (60), a critical assessment of pathogenicity predictions for SAVs at IDP regions might be an important future direction.

Overall, *Rhapsody-2* is a comprehensible tool that not only distinguishes pathogenic and neutral mutations, but also provides insights into the biophysical basis of disease-causing mutations. Its ability to integrate and analyze diverse data is essential to gaining a deeper understanding of the impact of mutations and providing guidance toward developing more effective therapies.

## Materials and Methods

Detailed descriptions of the datasets, features, XGBoost classifier specifications, and performance metrics used in this study are presented in the SI-Appendix. The datasets and codes are available at https://github.com/anupam-banerjee/rhapsody-2.

## Supplementary Material

Supplement 1

## Figures and Tables

**Table 1.** Comparison of the input and performance of the two generations of pathogenicity predictors, *Rhapsody* and *Rhapsody-2 *

Data/method	*Rhapsody*	*Rhapsody-2*	Fold increase
# of SAVs ^1^	20,361	117,525	5.8x
# of proteins	2,423	12,094	5x
ML method	Random Forest	XGBoost	
# of features	10	100	10x
AUROC ^2^	0.83	0.97 (0.94)	1.2x

1 All SAVs have at least one ClinVar review star and no conflicting reports

2 AUROC values are based on 10-fold stratified cross-validation. The value in parenthesis refers to 10-fold protein-stratified cross-validation, i.e. that obtained upon excluding all SAVs corresponding to the tested protein from the training dataset.

## Significance Statement

Understanding the impact of single amino acid variants (SAVs) on protein function is pivotal for detecting and treating genetic disorders. We present *Rhapsody-2,* a machine learning tool that leverages AlphaFold2-predicted protein structures to accurately predict the pathogenicity of SAVs. By integrating sequence, structural, and dynamic properties, *Rhapsody-2* achieves a high performance in distinguishing pathogenic variants across multiple benchmark datasets. Our tool not only provides a robust framework for predicting the impact of SAVs but also allows for mechanistic interpretability of pathogenicity, offering a valuable resource for understanding genotype-to-phenotype relationships toward assisting in precision medicine.
